# Supplementary material for: Nuclear condensates of p300 formed though the structured catalytic core can act as a storage pool of p300 with reduced HAT activity
Source: Nat Commun. 2021 Jul 29;12:4618. doi: 10.1038/s41467-021-24950-8 (PMC8322156; doi:10.1038/s41467-021-24950-8)
Supplement: Supplementary file 1 — Supplementary Info [file 41467_2021_24950_MOESM1_ESM.pdf]

Supplementary Information

Nuclear condensates of p300 formed though the structured catalytic core can act as a storage pool of p300 with reduced HAT activity

Yi Zhang, et al.

YFP-p300$_{BRPHZT}$

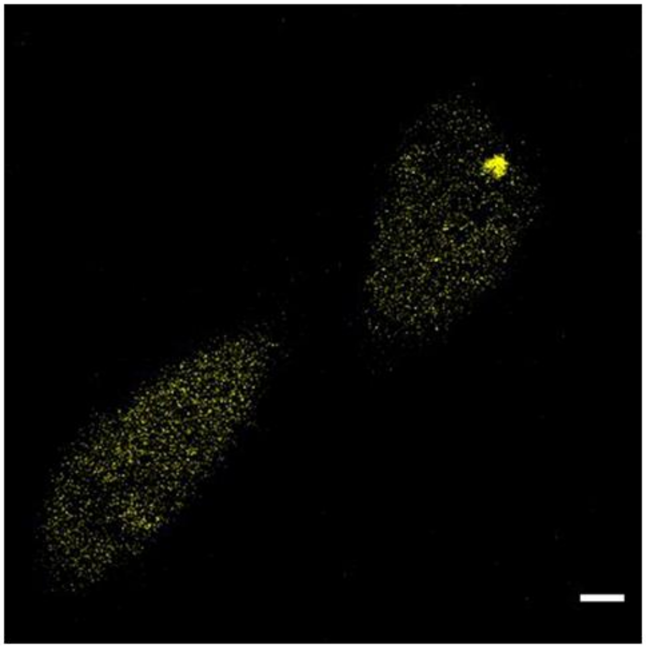

Supplementary Figure 1. p300$_{BRPHZT}$ phase separates to condensates in living cells. Representative views of HeLa cells (from at least three replicates) expressing YFP-p300$_{BRPHZT}$. Scale bar, 5 µm. Related to Figure 1.

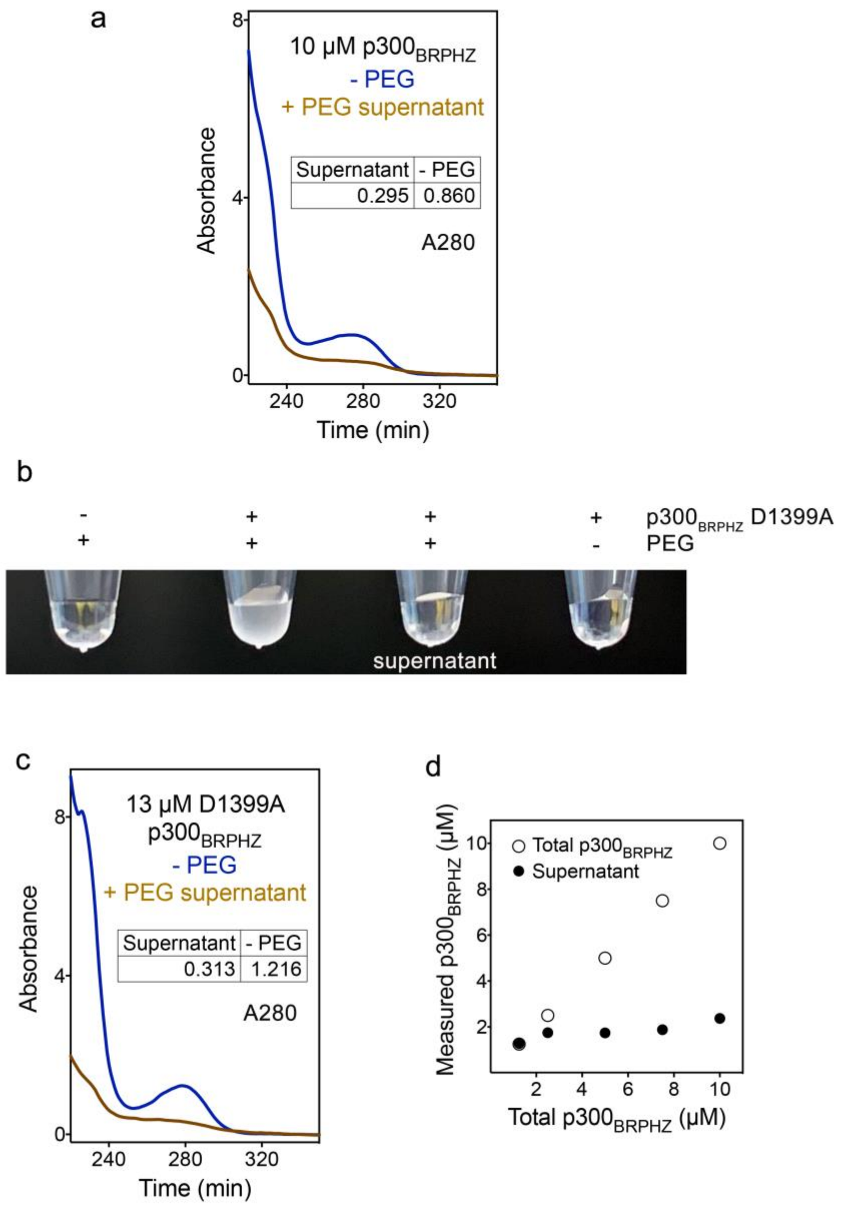

Supplementary Figure 2. Condensates have higher concentration of p300$_{BRPHZ}$ than supernatant. (a) UV absorbance of SIRT2-deacetylated WT p300$_{BRPHZ}$ in solution (dark blue) and of supernatant after droplets of the phase separated p300$_{BRPHZ}$ + PEG sample were spun down (brown). (b) Phase separation of D1399A p300$_{BRPHZ}$. The mixture contained 13 μM p300$_{BRPHZ}$ and 12% PEG. After centrifugation of the sample containing p300$_{BRPHZ}$ condensates, the supernatant became clear. (c) UV absorbance of D1399A p300$_{BRPHZ}$ in solution (dark blue) and of supernatant after droplets of the phase separated D1399A p300$_{BRPHZ}$ + PEG sample were spun down (brown). (d) p300$_{BRPHZ}$ samples containing 12% PEG were prepared at indicated concentration (open circles). Droplets formation was confirmed by imaging for each sample. No droplets were observed at the lowest concentration of 1.2 μM. The critical concentration was measured via absorbance at 280 nm of the supernatant (filled circles) after removing the droplets by centrifugation. Related to Figure 1.

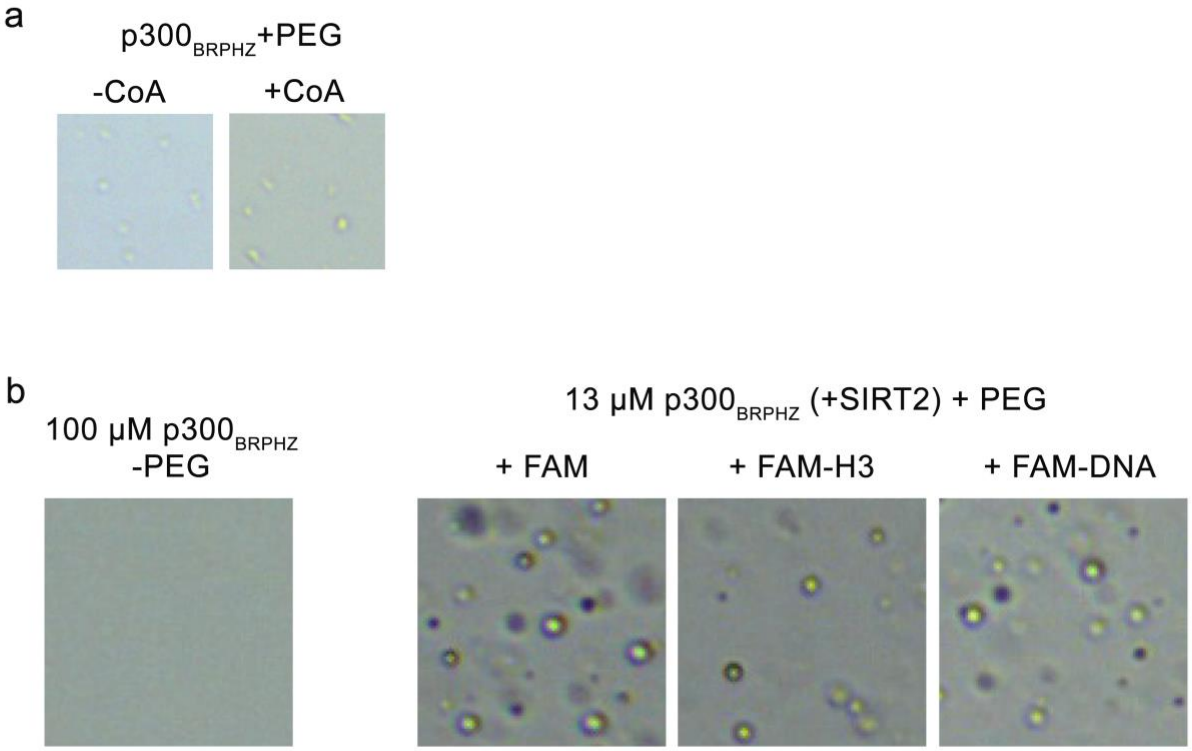

Supplementary Figure 3. Phase separation of SIRT2-treated p300$_{BRPHZ}$. (a) Representative images of p300$_{BRPHZ}$ samples (incubated with/without 1 mM CoA) under microscope. A 50 µm × 50 µm square region is shown for each sample. (b) Representative images of phase separated SIRT2-treated p300$_{BRPHZ}$ (13 µM) condensates with FAM, FAM-labeled H3 and FAM-labeled DNA. A 50 µm × 50 µm square region is shown for each sample. p300$_{BRPHZ}$ (100 µM) does not phase separate without PEG (left). Experiments in a-b were performed in at least two replicates. Related to Figures 3 and 4.

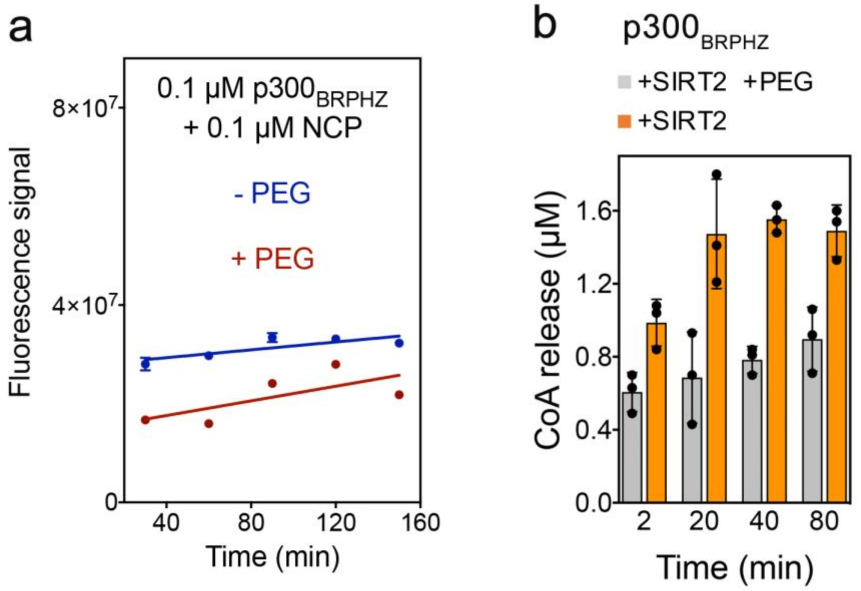

Supplementary Figure 4. HAT activity of $p300_{BRPHZ}$ in dilute solution and droplet suspensions. (a) HAT activity of diluted (0.1 μM) SIRT2-deacetylated $p300_{BRPHZ}$ +/- PEG on NCP (0.1 mM) was measured by fluorometric assay. The relative reactive rates (slope values) are 0.003 (-PEG) and 0.006 (+PEG). Data are presented as mean values +/- S.D.; error bars represent S.D. from triplicate measurements. (b) HAT activity of SIRT2-treated $p300_{BRPHZ}$ in dilute solution (orange, as in Figure 3a) or droplet suspensions (grey) measured by fluorometric assay. Reactions were started by addition of acetyl-CoA and quenched by flash-freeze at indicated time points. Data are presented as mean values +/- S.D.; error bars represent S.D. from triplicate measurements. Related to Figure 6.

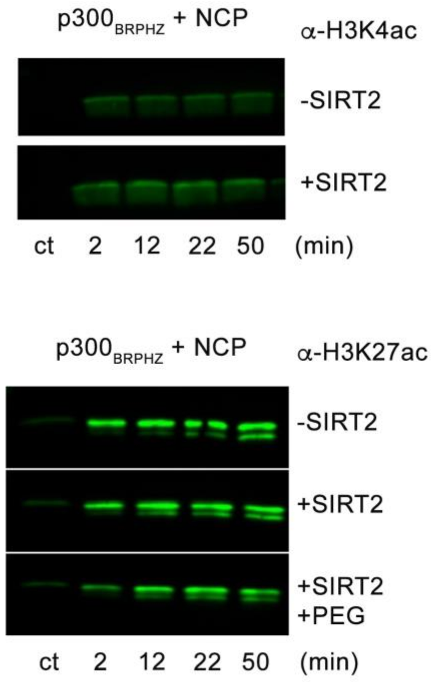

Supplementary Figure 5. HAT activity of $p300_{BRPHZ}$ on histone H3 in nucleosomes. H3K4ac and H3K27ac western blot analysis of the reaction mixtures containing $p300_{BRPHZ}$ and equimolar amount of NCP as a substrate. Reactions were quenched by rapid freezing and addition of SDS-loading buffer at indicated time points. Experiments were performed twice for H3K27ac and once for H3K4ac. Related to Figure 7.
